# Usefulness of Strain Echocardiography in Heart Failure with Preserved Ejection Fraction

**DOI:** 10.3390/jcm15166377

**Published:** 2026-08-18

**Authors:** Maria Concetta Pastore, Clarissa Carmona De Azevedo Bellagamba, Andrea Stefanini, Alessia Pinelli, Giulia Elena Mandoli, Luna Cavigli, Flavio D’Ascenzi, Marta Focardi, Matteo Cameli

**Affiliations:** Department of Medical Biotechnologies, Division of Cardiology, University of Siena, 53100 Siena, Italy

**Keywords:** heart failure with preserved ejection fraction, speckle-tracking echocardiography, left ventricle global longitudinal strain, atrial strain, right ventricular strain, diagnosis

## Abstract

Heart failure with preserved ejection fraction (HFpEF) represents an increasingly prevalent clinical syndrome, driven by population ageing and the growing burden of cardiometabolic comorbidities, and is associated with significant morbidity and mortality. Due to its heterogeneous pathophysiology and the frequent absence of overt structural abnormalities, early diagnosis and accurate risk stratification remain challenging. In the current era of emerging disease-modifying therapies, the identification of sensitive imaging markers able to detect early myocardial dysfunction and refine patient characterization has become increasingly important. Speckle-tracking echocardiography has emerged as a valuable tool for the comprehensive evaluation of HFpEF, allowing the assessment of subclinical myocardial impairment beyond conventional parameters. Left ventricular global longitudinal strain (LV-GLS) identifies subtle systolic dysfunction despite preserved left ventricular ejection fraction (LVEF) and provides incremental diagnostic and prognostic information. Accordingly, LV-GLS has been incorporated into contemporary diagnostic algorithms and may represent a promising marker for monitoring disease progression and therapeutic response. Beyond the left ventricle (LV), left atrial (LA) strain has gained increasing relevance as a marker of atrial myopathy and elevated filling pressures. Left atrial reservoir strain (LARS) detects early atrial dysfunction before overt structural remodelling, improves the identification of HFpEF in patients with unexplained dyspnoea, and provides additional prognostic information, including prediction of atrial fibrillation and thromboembolic risk. Moreover, right ventricular free-wall longitudinal strain (RV-FWLS) allows early recognition of right ventricular involvement and has shown important prognostic implications, particularly in relation to pulmonary vascular dysfunction and exercise intolerance. Overall, a multi-chamber strain-based approach may improve HFpEF diagnosis, phenotyping, and risk stratification, supporting a transition toward a more personalized management strategy. Further prospective studies are needed to define the role of strain imaging in guiding therapeutic decisions and monitoring treatment response.

## 1. Introduction

Heart Failure (HF) is a clinical syndrome consisting of cardinal symptoms such as breathlessness and fatigue that may be accompanied by signs such as elevated jugular venous pressure, pulmonary crackles and peripheral edema due to structural and/or functional cardiac abnormalities resulting in decreased cardiac output and/or heightened intracardiac diastolic pressures [1].

HF is divided into distinct phenotypes based on the measurement of the LVEF: those with an LVEF ≤ 40% are defined as having HF with reduced ejection fraction (HFrEF), while an LVEF between 41% and 49% is designated as HF with mildly reduced ejection fraction (HFmrEF). Patients presenting with an LVEF ≥ 50% are classified as HFpEF. This classification guides therapeutic strategy, as the efficacy of treatments in reducing mortality and HF-related hospitalization varies across different HF phenotypes.

Over the past decades, HFpEF has become increasingly prevalent, currently accounting for nearly half of all HF cases and is associated with significant morbidity and mortality. Its incidence is particularly high among elderly individuals and patients with hypertension, diabetes mellitus, obesity, atrial fibrillation, and chronic kidney disease, considering the total burden of HFpEF is projected to become the dominant HF subtype in the future, affecting approximately 1 in 10 adults during their lifetime. Therefore, clinicians should always consider HFpEF as a potential diagnosis in patients with dyspnoea or exercise intolerance [2].

LVEF is fundamentally a volumetric measurement driven primarily by radial and circumferential thickening of the epicardial myocardial layers. In HFpEF, these outer layers routinely maintain compensatory function, masking advanced subendocardial disease. Because the subendocardium is predominantly composed of longitudinal fibres that are highly sensitive to ischemia, microvascular stress, and hypertrophy, longitudinal contraction is frequently impaired long before gross volumetric changes occur. Relying solely on conventional LVEF therefore creates a diagnostic blind spot, underestimating true dysfunction [3,4]. 

At the cellular level, the systemic comorbidities characteristically clustering in HFpEF, such as obesity, type 2 diabetes, and metabolic syndrome, drive a chronic, low-grade systemic inflammatory state. This sustained inflammation triggers coronary microvascular endothelial dysfunction, reducing nitric oxide bioavailability and downregulating protein kinase G signalling in adjacent cardiomyocytes, which promotes hypertrophy. Concurrently, activated circulating fibroblasts transdifferentiate into myofibroblasts, accelerating the interstitial deposition of collagen and advanced glycation end-products within both the ventricular and atrial extracellular matrices. Within the LA, this accelerated myocardial fibrosis directly drives an acute elevation in LA stiffness and a profound loss of tissue compliance. This metabolic-inflammatory cascade explains why mechanical abnormalities in the LA develop early in the disease, manifesting as a premature drop in LA strain parameters well before structural dilation or traditional diastolic Doppler abnormalities become visible on standard imaging.

So, in addition to left ventricular assessment, strain imaging has expanded the evaluation of atrial and right ventricular (RV) function. LA strain has demonstrated utility in estimating LV filling pressures and evaluating diastolic dysfunction (DD) [5], while right ventricular strain has shown prognostic importance in patients with pulmonary hypertension and HFpEF [6,7]. Furthermore, strain echocardiography has been associated with exercise intolerance, hospitalization risk, and overall cardiovascular outcomes, highlighting its potential role in risk stratification and disease monitoring. 

While several excellent reviews have previously detailed the diagnostic criteria for HFpEF, existing literature frequently treats strain imaging as an isolated, single-chamber tool. A comprehensive framework that integrates LV, LA, and RV strain into a synchronized, multi-chamber paradigm remains a notable gap in the current literature. This review addresses this critical gap and establishes its innovative positioning by systematically decoupling the utility of multi-chamber strain into distinct diagnostic, prognostic, and therapeutic-monitoring domains, providing clinicians with a highly structured, actionable guide for personalized HFpEF management. In this light, the aim of this paper is to review the principles of strain echocardiography and discuss its diagnostic, risk stratification, prognostic, and clinical usefulness in patients with HFpEF, with particular attention to its role in identifying subclinical myocardial dysfunction and guiding patient evaluation. We have conducted a comprehensive literature search in the PubMed, Scopus, and Web of Science databases to identify relevant studies on the utility of strain analysis in HFpEF using MeSH and free-text keywords, including: “advanced echocardiography”, “speckle tracking”, “strain”, and “HFpEF”. The search was limited to articles published in the English language.

## 2. Left Ventricular Strain in HFpEF

### 2.1. Left Ventricular Strain for HFpEF Diagnosis

Although HFpEF has traditionally been considered a disorder primarily characterized by DD, increasing evidence suggests that impaired systolic myocardial mechanics are also involved in its pathophysiology, and several studies have demonstrated the presence of subtle systolic dysfunction in HFpEF patients that cannot be detected using conventional echocardiographic parameters alone [8].

LVEF measures the percentage of blood ejected from the ventricle during systole and depends mainly on changes in ventricular volume. In HFpEF, the preservation of LVEF is partly explained by compensatory circumferential and radial myocardial contraction despite impairment of the subendocardial longitudinal fibres, which are particularly susceptible to fibrosis, microvascular ischemia, hypertrophy, and increased wall stress [4,9]. As a result, longitudinal systolic dysfunction may develop early in the disease process despite preserved LVEF [10]. LV-GLS primarily evaluates the function of the subendocardial longitudinal myocardial fibres. During systole, these fibres shorten longitudinally from the base toward the apex of the heart. LV-GLS quantifies this deformation as a percentage change in myocardial length relative to its original length.

Speckle Tracking Echocardiography (STE) overcomes the limitations of conventional LVEF assessment by tracking natural acoustic markers (“speckles”) within the myocardium. This allows for an angle-independent, quantitative measurement of actual myocardial deformation, providing a highly sensitive evaluation of ventricular mechanics through strain analysis [11]. Moreover, LV-GLS has shown higher reproducibility compared to LVEF, as shown in the Mevedovsky study: repeated LV-GLS measurements produced an interclass correlation coefficient = 0.98, while LVEF = 0.89. Moreover, the agreement between experienced and non-experienced readers was also better for LV-GLS (r = 0.98) than LVEF (r = 0.91), with smaller bias for LV-GLS [8].

**Figure 1:**
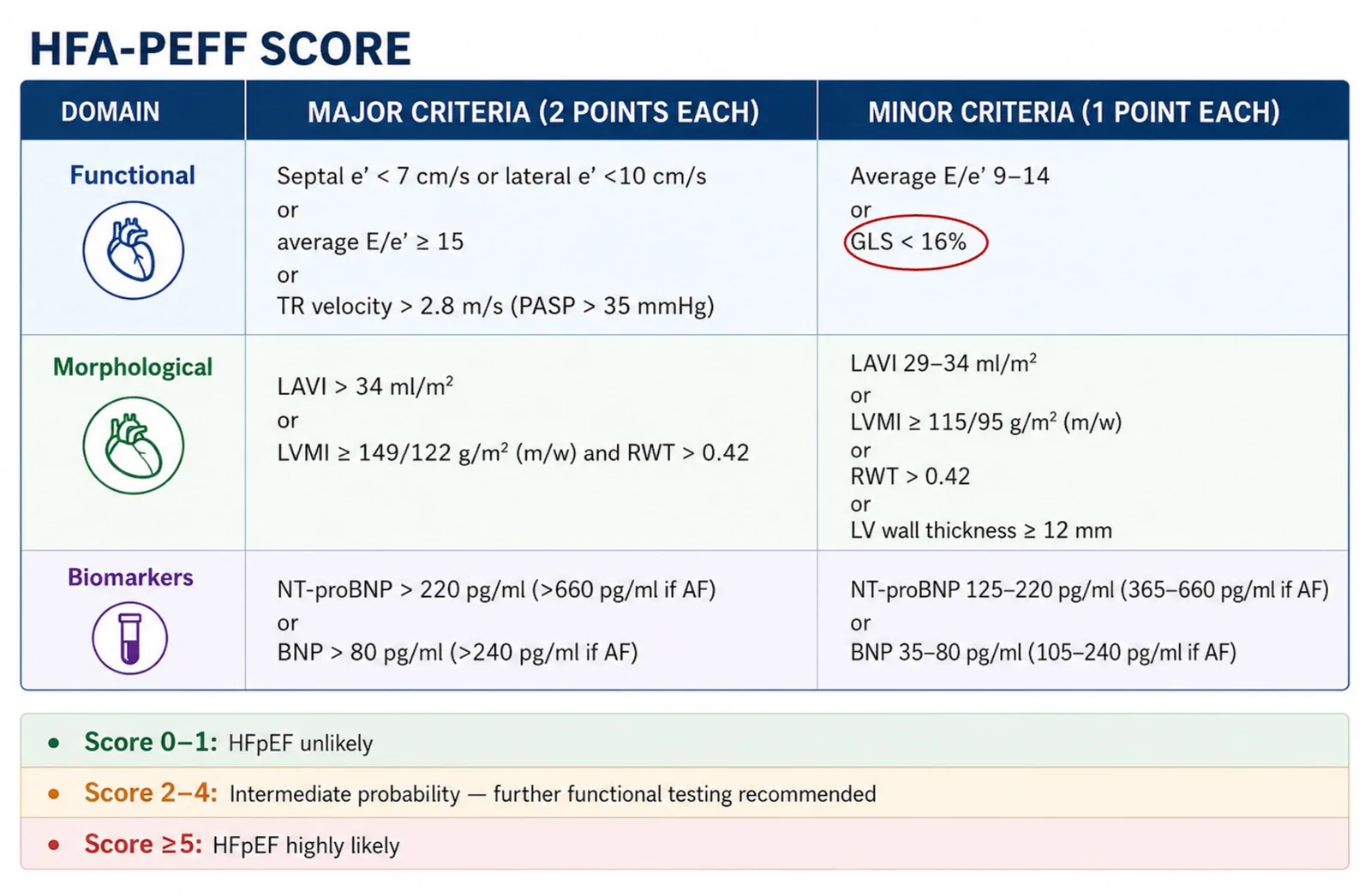
HFA-PEFF score. e’, early diastolic mitral annular velocity; E, early transmitral flow velocity; TR V, tricuspid regurgitation velocity; PASP, pulmonary artery systolic pressure; GLS, left ventricular global longitudinal strain; LAVI, left atrial volume index; LVMI, left ventricular mass index; RWT, relative wall thickness; LV, left ventricle; NT-proBNP, N-terminal pro-B-type natriuretic peptide; BNP, B-type natriuretic peptide; AF, atrial fibrillation. NT-proBNP should be interpreted according to age-specific cut-off values: ≥125 pg/mL, ≥250 pg/mL and ≥500 pg/mL for <50 years, 50–74 years and ≥75 years, respectively [13].

Morris et al., in a meta-analysis of patients with HFpEF, showed that impaired longitudinal systolic function is a consistent finding in this population, supporting the concept that HFpEF is associated with subclinical systolic dysfunction [11]. The European Society of Cardiology Heart Failure Association (HFA)-PEFF diagnostic scoring system (Figure 1) recognizes the LV-GLS as an important marker of DD and elevated filling pressures that can improve diagnostic accuracy [12].

Furthermore, LV-GLS is a critical clinical tool for distinguishing primary HFpEF with ventricular hypertrophy from underlying phenocopies, such as hypertrophic cardiomyopathy (HCM), Fabry disease (FD), and cardiac amyloidosis (CA). While all these conditions may present clinically as HFpEF with an absolute reduction in LV-GLS, their regional strain patterns differ distinctly. In primary HFpEF, typically driven by comorbidities like hypertension, metabolic syndrome, and ageing, the impairment in LV-GLS is generally diffuse and global, without an evident regional gradient between the base and the apex. On the other hand, CA demonstrates a classic “apical sparing” pattern, where the basal and mid-ventricular segments show profoundly depressed strain while the apex is preserved. This visual “cherry-on-top” pattern can be quantified using the Relative Apical Sparing Ratio (RASR = average apical strain/[average basal + mid-ventricular strain]). A ratio greater than 1 is highly suggestive of CA over classic HFpEF or other forms of hypertrophy. HCM is characterized by a localized, segmental impairment of LV-GLS that corresponds directly to regional areas of myofibrillar disarray and hypertrophy. While FD characteristically demonstrates early, localized strain impairment in the basal posterolateral wall, tracking precisely with the site of early myocardial fibrosis (Table 1).

Additionally, global LV myocardial work (MW) indexes derived from non-invasive LV pressure/LV-GLS loop analysis, specifically global work index (GWI), can be useful as an additional discriminative value over LV-GLS alone in differentiating CA and HCM, as demonstrated by de Gregorio et al. [14]. In another study, the same group demonstrated that a substantial percentage of patients with HCM and CA present impaired systemic vascular resistance (SVR) and/or GWI, despite preserved LVEF [15], which can further help to distinguish hypertrophic phenocopies.

### 2.2. Left Ventricular Strain for HFpEF Risk Stratification and Prognosis

LV-GLS has emerged not only as a diagnostic tool but also as an important parameter for risk stratification in patients with HFpEF. Reduced LV-GLS has also been correlated with elevated left ventricular filling pressures, impaired exercise capacity, degree of myocardial fibrosis, worsening symptoms and although LVEF remains preserved in these patients, several studies have demonstrated that impaired myocardial deformation assessed by STE is associated with worse clinical outcomes, suggesting that LV-GLS reflects the true severity of myocardial dysfunction more accurately than conventional systolic measurements and reflects the extent of myocardial remodelling and increased stiffness characteristic of HFpEF [10,11]. A 2025 prospective study of 335 patients with HFpEF demonstrated that 2D speckle-tracking GLS independently predicted adverse outcomes over a median 17.6-month follow-up (HR 1.323 per 1% worsening; 95% CI 1.225–1.429; *p* < 0.001). Importantly, adding GLS to the clinical model improved prognostic discrimination (C-index 0.719) and significantly enhanced risk reclassification (net reclassification index, NRI 0.612; both *p* < 0.001), supporting the incremental prognostic value of GLS beyond conventional risk factors [16].

Moreover, GLS has demonstrated incremental prognostic value beyond traditional risk markers such as LVEF, LA volume, and Doppler diastolic indices or even natriuretic peptide levels [17]. Incorporation of strain imaging into routine echocardiographic evaluation may therefore improve patient stratification and assist clinicians in identifying individuals who require closer follow-up and more aggressive therapeutic management.

### 2.3. Left Ventricular Strain for HFpEF Therapeutic-Monitoring

LV strain mechanics are increasingly utilized to track myocardial responses to therapeutic interventions in HFpEF. Serial evaluations of LV-GLS provide a sensitive means of monitoring reverse myocardial remodelling and therapeutic efficacy. Specifically, recent clinical trial data demonstrate that guideline-directed medical therapies, most notably SGLT2 inhibitors and intensive neurohormonal modulation, as well as structured lifestyle and weight-loss interventions, can arrest or partially reverse subendocardial longitudinal dysfunction, manifesting as measurable improvements in LV-GLS over time [18,19,20].

## 3. Left Atrial Strain in HFpEF

### 3.1. Diagnostic Role of Left Atrial Strain in HFpEF

#### 3.1.1. Technical and Clinical Principles

LA strain has emerged as a valuable echocardiographic parameter in the evaluation of HFpEF. The LA plays a central role in modulating LV filling through its reservoir, conduit, and booster pump functions. In HFpEF, chronic elevation of LV filling pressure leads to progressive atrial remodelling, fibrosis, and functional impairment, which may occur before significant atrial enlargement becomes evident [21,22] and may precede the reduction in LV-GLS, highlighting the role of LA strain as an early and sensitive marker that should be incorporated in the comprehensive echocardiographic evaluation of patients with suspected HFpEF. STE allows quantitative assessment of atrial myocardial deformation and provides a sensitive marker of atrial dysfunction.

**Figure 2:**
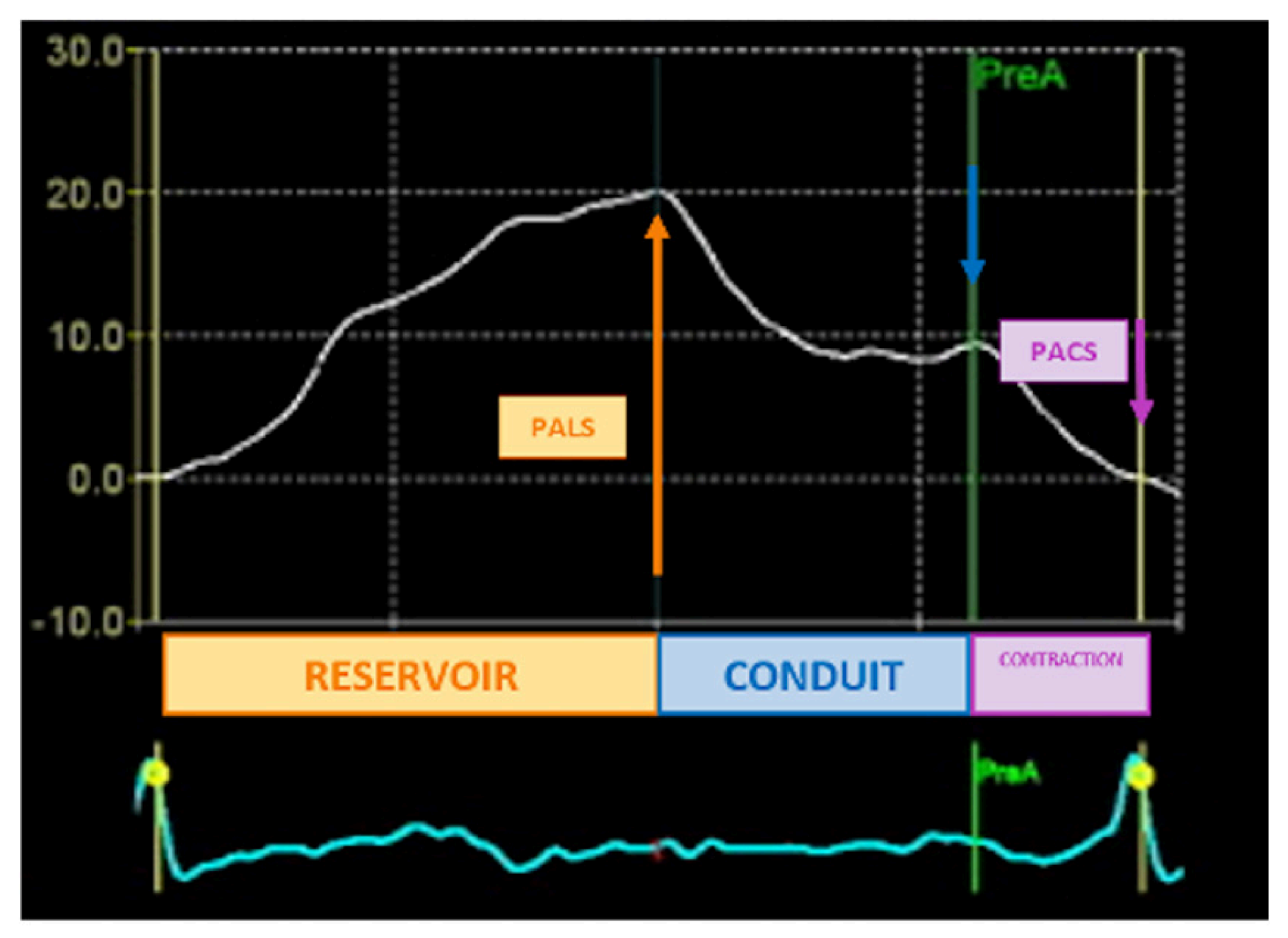
Phases of left atrial strain. PALS, peak atrial longitudinal strain; PACS, peak atrial contraction strain.

In the reservoir phase, as the LA fills and stretches, there is positive atrial strain that reaches its peak in LV systole at the end of LA filling, prior to opening of the mitral valve; this corresponds to the LA reservoir strain (LARS) or peak atrial longitudinal strain (PALS), which is measured at the end of the reservoir phase. It evaluates the ability of LA to expand, reflecting its compliance. Following this, passive LA emptying ensues with opening of the mitral valve, resulting in decreased atrial strain with negative deflection of the strain curve up to a plateau period, which is analogous to diastasis; this corresponds to the LA conduit strain. A second deflection in the strain curve is then observed corresponding to atrial systole, corresponding to the peak atrial contraction strain (PACS) or late diastolic strain, is measured following the P wave and corresponds to active atrial contraction, reflecting atrial pump function and contractility [22] (Figure 2).

**Figure 3:**
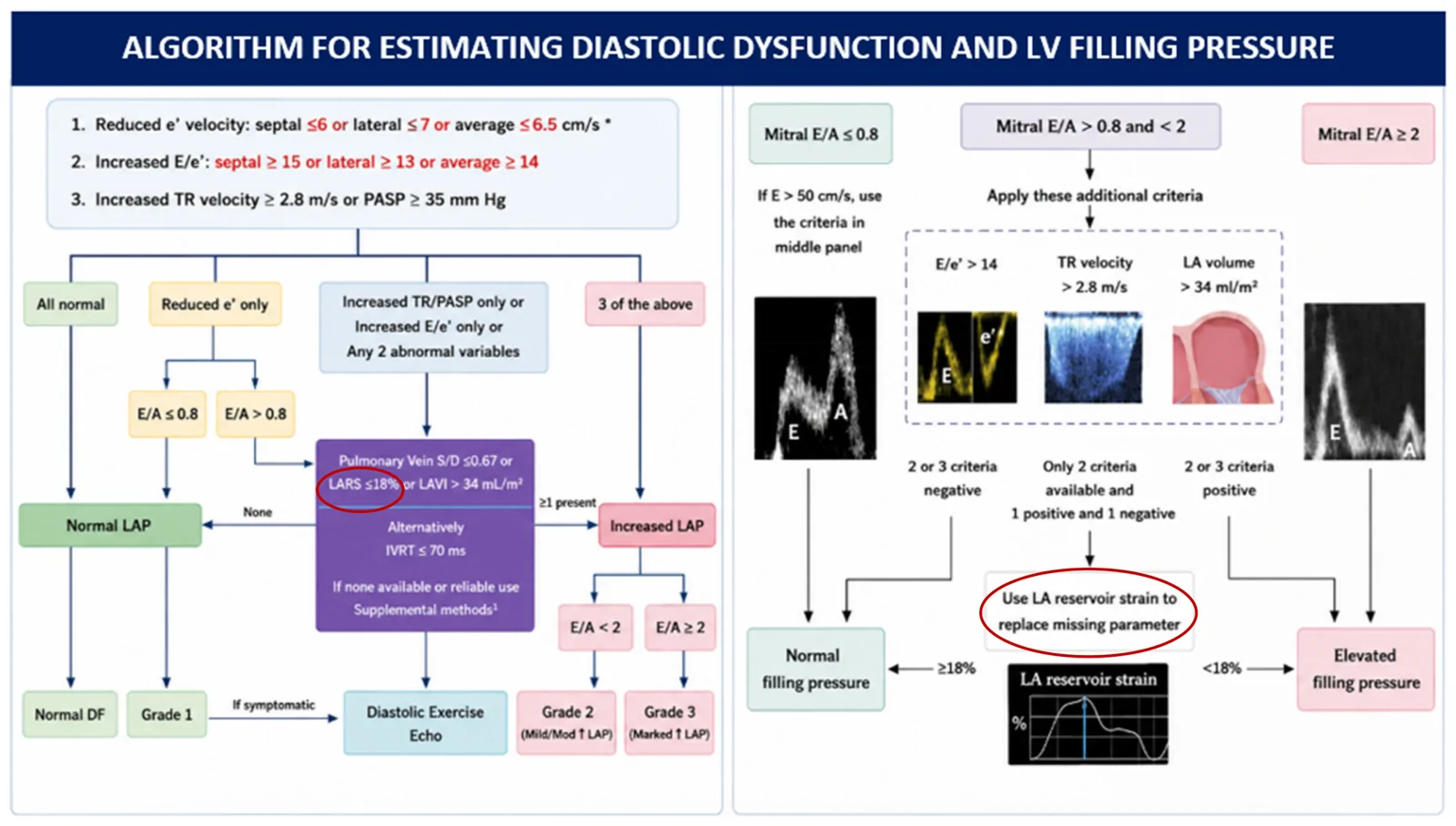
American Society of Echocardiography (ASE) and European Association of Cardiovascular Imaging (EACVI) algorithms for estimating diastolic dysfunction and LV filling pressure. DF, diastolic function; E, early diastolic transmitral flow velocity; E/A, ratio between early and late diastolic transmitral flow velocities; e′, early diastolic mitral annular velocity assessed by tissue Doppler imaging; E/e′, ratio between early diastolic transmitral flow velocity and early diastolic mitral annular velocity; IVRT, isovolumic relaxation time; LA, left atrial; LAP, left atrial pressure; LARS, left atrial reservoir strain; LAVI, left atrial volume index; LV, left ventricular; PASP, pulmonary artery systolic pressure; TR, tricuspid regurgitation velocity. * in the presence of satisfactory septal and lateral e’ velocity, the average e’ velocity and E/E’ ratio are relied on, in case of absent or suboptimal septal or lateral velocity, then the satisfactory signal is relied on along with the corresponding E/e’ rato.

Among atrial strain parameters, PALS has shown the strongest clinical utility, reflecting atrial compliance and the interaction between atrial and ventricular function. Reduced LA strain has been associated with elevated filling pressures, impaired diastolic reserve, pulmonary hypertension, reduced exercise capacity, atrial fibrillation (AF), and adverse cardiovascular outcomes in HFpEF patients [23,24] and, for this reason, has been added in the latest recommendations for the evaluation of DD and HFpEF [25,26] (Figure 3).

#### 3.1.2. Left Atrial Strain in Patients with Risk Factors for HFpEF

LA dysfunction may develop early in patients with cardiovascular risk factors strongly associated with HFpEF, including hypertension, diabetes mellitus, obesity, chronic kidney disease, and metabolic syndrome. In these patients, chronic exposure to increased afterload, systemic inflammation, myocardial fibrosis, and microvascular dysfunction promotes progressive impairment of atrial compliance and reservoir function [27].

Several studies have demonstrated that reduced LA strain can identify subclinical DD even before overt HFpEF develops. In hypertensive and diabetic patients, impaired PALS correlates with increased LV stiffness and elevated filling pressures despite preserved LVEF and normal atrial size [28,29]. Similarly, obesity-related myocardial remodelling has been associated with early atrial mechanical dysfunction detectable by strain imaging, even in asymptomatic patients. Reduced PALS and PACS have been observed in overweight and obese individuals, suggesting that atrial functional impairment may precede overt structural remodelling and contribute to the development of HFpEF [30,31].

Therefore, LA strain may represent an early marker of atrial remodelling and a useful tool for identifying patients at increased risk of developing HFpEF before structural abnormalities become apparent on conventional echocardiography.

#### 3.1.3. Left Atrial Strain Internationally Recommended as Diagnostic Parameter in HFpEF

Growing evidence supporting the diagnostic value of LA strain has led to its inclusion in international recommendations for DD and HFpEF evaluation, such as the Lababidi et al. algorithm [25], EACVI algorithm [26], as well as the recommendations of the British Society of Echocardiography [20]. Traditional echocardiographic markers such as LA volume index and E/e′ ratio may be insufficiently sensitive in early disease stages or may provide inconclusive results in intermediate-risk patients. In contrast, PALS directly reflects atrial mechanical dysfunction secondary to chronically elevated LV filling pressure [32].

**Figure 4:**
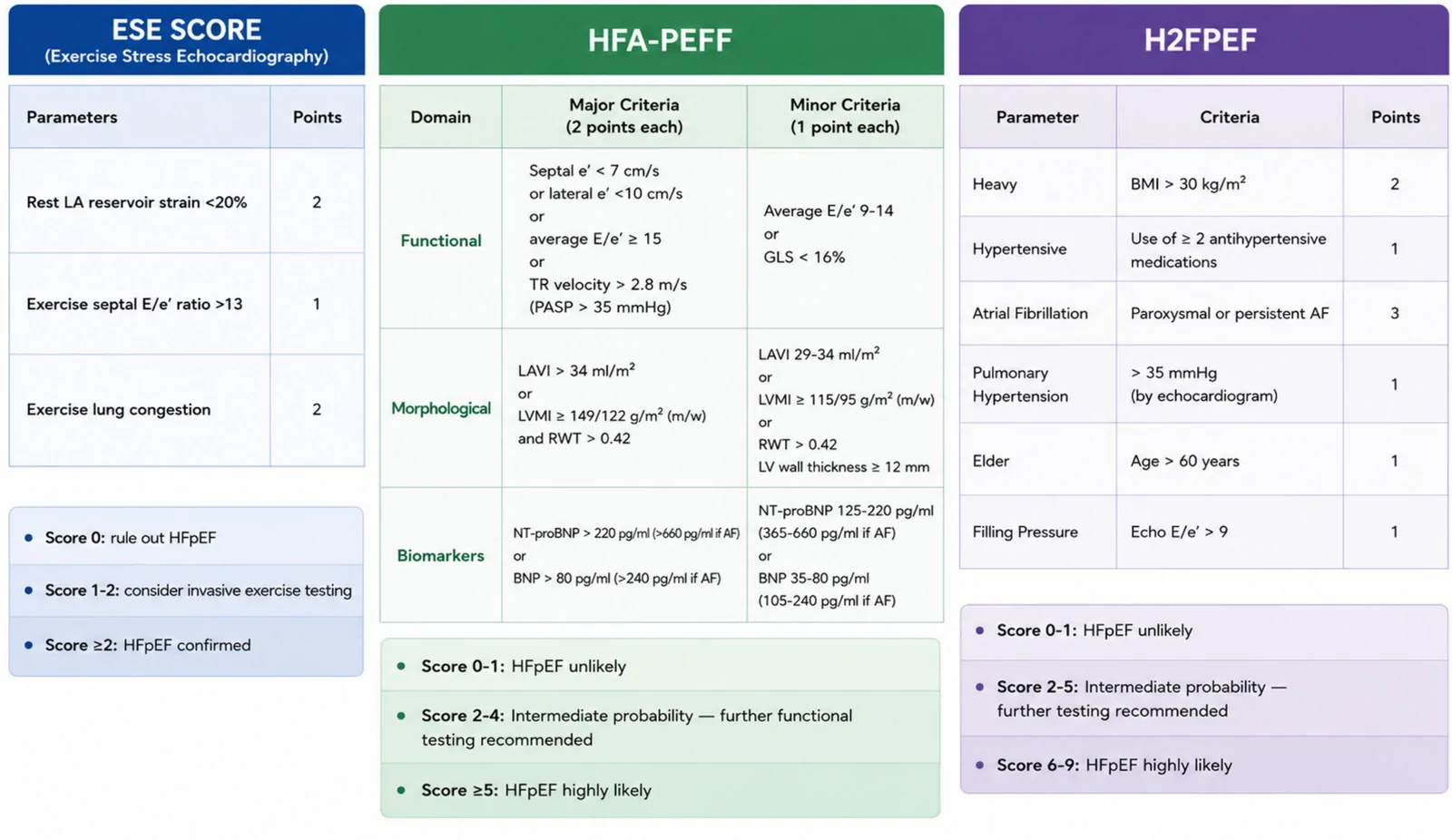
Comparison of the ESE score, HFA-PEFF and H2FPEF and the use of STE evaluation for patients at risk of HFpEF. AF, atrial fibrillation; BMI, body mass index; BNP, B-type natriuretic peptide; E, early diastolic transmitral flow velocity; E/e′, ratio between early transmitral flow velocity and early diastolic mitral annular velocity; ESE, exercise stress echocardiography; GLS, global longitudinal strain; HFpEF, heart failure with preserved ejection fraction; HFA-PEFF, Heart Failure Association Pre-test assessment, Echocardiography and natriuretic peptide score, Functional testing, Final etiology score; H2FPEF, Heavy, Hypertensive, Atrial Fibrillation, Pulmonary Hypertension, Elder, Filling Pressure score; LA, left atrial; LAP, left atrial pressure; LARS, left atrial reservoir strain; LAVI, left atrial volume index; LV, left ventricular; LVMI, left ventricular mass index; NT-proBNP, N-terminal pro–B-type natriuretic peptide; PASP, pulmonary artery systolic pressure; RWT, relative wall thickness; TR, tricuspid regurgitation.

Reduced PALS has demonstrated a strong correlation with invasively measured pulmonary capillary wedge pressure and exercise hemodynamics, supporting its role as a non-invasive marker of LV DD, as demonstrated by Backhaus et al. [33]. Kagami et al. created a score system for diagnosing HFpEF based on three echocardiographic variables. They demonstrated that rest PALS <20%, exercise septal E/e’ ratio >13 and exercise lung congestion evaluated by increases in ultrasound B-lines are independent predictors of HFpEF. A weighted score was created with these variables (the ESE score) ranging from 0 to 5. The ESE score accurately discriminated HFpEF from controls and classified the HFpEF probability into three categories (probabilities: low risk 28%, intermediate risk 59–83%, and high risk 95–99%) [34] (Figure 4).

Moreover, LA function can be used as a tool to distinguish HFpEF with ventricular hypertrophy phenocopies, whereas it might be altered in primary HFpEF; it is significantly worse in HFpEF due to infiltrative disease, such as CA, despite a similar increase in LV wall thickness [35], as well as compared with HCM patients, as demonstrated by Monte et al. [36].

### 3.2. Left Atrial Strain for Prognostic Assessment in HFpEF

#### 3.2.1. Prognostic Value of Left Atrial Strain in HFpEF

The LA strain is not just a tool for diagnosis of HFpEF but also correlates with clinical events. Several studies have demonstrated the prognostic significance of LA strain in patients with HFpEF. A systematic review and meta-analysis by Khan et al., including more than 1900 HFpEF patients, showed that impaired PALS was significantly associated with increased risk of all-cause mortality and HF hospitalization. Reduced LA strain reflects chronic elevation of LV filling pressures, atrial remodelling, and myocardial fibrosis, suggesting that atrial dysfunction may represent an integrated marker of disease severity in HFpEF [37,38].

The PARAMOUNT trial utilized LA strain analysis to compare LA function in patients with HFpEF against healthy controls. The study revealed that HFpEF patients suffer from significant impairment across all phases of atrial activity: reservoir, conduit, and pump function. Notably, PALS remained significantly reduced in the HFpEF cohort even after adjusting for confounders, appearing even in patients with normal LA size and no history of AF. Furthermore, in HFpEF patients, lower LA strain parameters were associated with higher prevalence of prior HF hospitalization and history of AF, as well as lower LV systolic function, higher LV mass and LAVi [39].

In a study with HFpEF and HFmrEF patients presenting exercise intolerance, the reduction in PALS was a reliable marker of decreased peak exercise capacity, evaluated with maximal cardiopulmonary exercise test and classified according to peak VO2 cut-off of prognostic value in this group of patients [23]. Another study showed that impaired PALS during exercise in HFpEF patients is associated with biventricular limitations, exercise intolerance, and increased risks of HF events [22]. Furthermore, in a study evaluating patients presenting with exertional dyspnoea, Telles et al. performed simultaneous STE and invasive right heart catheterization during supine cycle ergometry. While PALS and PACS parameters demonstrated significant correlations with resting hemodynamics, their association was far more pronounced during exercise. Both PALS and PACS were strongly correlated with peak exercise pulmonary capillary wedge pressure (PCWP), with lower levels of PALS and PACS corresponding with higher pressures and LA stiffness was strongly related to B-type natriuretic peptide levels [40]. These findings underscore that exercise-based LA strain imaging serves as a superior dynamic marker capable of unmasking latent LA non-compliance and elevated LV filling pressures that remain clinically occult under resting conditions. 

Finally, the ESE score mentioned was used in a cohort of 620 dyspnoeic patients; the predictive ability of the derived score was assessed and a higher ESE score was associated with an increased risk of all-cause mortality or worsening HF events even after adjusting for confounders [34].

#### 3.2.2. Left Atrial Strain as Predictor of Atrial Fibrillation Occurrence in HFpEF

AF is highly prevalent in patients with HFpEF and represents one of the most important factors contributing to symptom worsening, hospitalization, exercise intolerance, and mortality. The chronically elevated LV filling pressures lead to progressive remodelling, fibrosis and electrical instability. PALS has demonstrated diagnostic and prognostic value across a wide spectrum of conditions. In the context of HFpEF, it has emerged as a sensitive marker of atrial mechanical dysfunction and important predictor of AF occurrence. In AF, reduced strain correlates with atrial fibrosis and left atrial appendage dysfunction, identifying patients at increased risk of arrhythmia recurrence and thromboembolism [41]. 

Among atrial strain parameters, PALS has demonstrated the strongest association with AF development. Reduced PALS reflects impaired atrial compliance and increased atrial stiffness secondary to chronic pressure overload and myocardial fibrosis. Importantly, atrial mechanical dysfunction may occur before significant atrial enlargement becomes evident on conventional echocardiography, making LA strain a more sensitive indicator of early atrial remodelling. Olsen et al. demonstrated that PALS and PACS are independent predictors of subclinical AF in elderly at risk of stroke, independent of atrial size and diastolic function [42]. Obokata et al., in patients with AF, demonstrated that reduced PALS independently predicts embolic events and provides incremental prognostic value over CHA_2_DS_2_-VASc score [43]. These findings suggest that LA mechanical dysfunction may better reflect the underlying atrial substrate responsible for adverse outcomes.

The prognostic importance of LA strain extends beyond first AF occurrence. In patients with established AF, impaired LA strain has also been associated with greater risk of arrhythmia recurrence after cardioversion or catheter ablation, suggesting that reduced atrial deformation reflects advanced structural remodelling and irreversible atrial myocardial damage [44,45]. Consequently, assessment of LA strain may help identify HFpEF patients at higher arrhythmic and cardioembolic ischaemic stroke risk who could benefit from closer rhythm monitoring and earlier therapeutic intervention.

### 3.3. Left Atrial Strain in HFpEF for Therapeutic-Monitoring

Given its high sensitivity to changes in LV filling pressures, LA strain is an ideal parameter for tracking therapeutic response and reverse atrial remodelling. Effective clinical management, including intensive volume optimization, aggressive blood pressure control, and the initiation of guideline-directed medical therapies, relieves the chronic pressure overload imposed on the LA. Serial monitoring of PALS allows clinicians to objectively evaluate the stabilization or regression of atrial mechanical dysfunction, providing an early physiologic signal of therapeutic efficacy prior to any detectable alterations in gross structural volumes.

## 4. Right Ventricular Strain in HFpEF

### 4.1. Diagnostic Role of RV Strain in HFpEF

Although HFpEF has traditionally been considered a left-sided cardiac disorder, increasing evidence demonstrates that RV dysfunction frequently occurs during disease progression and is strongly associated with symptom severity, pulmonary hypertension, exercise intolerance, and adverse clinical outcomes [46].

In HFpEF, chronically elevated LV filling pressures lead to increased LA pressure and pulmonary venous congestion, eventually causing pulmonary vascular remodelling and pulmonary hypertension. The increase in RV afterload contributes to progressive RV remodelling and dysfunction, as evidenced in a rat model of obesity-associated HFpEF from Hubesch et al. [47]. Conventional echocardiographic parameters such as tricuspid annular plane systolic excursion (TAPSE) and fractional area change (FAC) may fail to detect early or subtle RV impairment because of the complex geometry of the right ventricle and the load dependency of these measurements [48,49].

STE allows quantitative assessment of RV myocardial deformation and provides a more sensitive evaluation of RV systolic function through RV longitudinal strain analysis. Since the right ventricle is predominantly composed of longitudinal myocardial fibres [50], RV-FWLS has demonstrated its utility for identifying subclinical RV dysfunction.

Furthermore, RV strain-derived metrics have diagnostic utility in characterizing RV–pulmonary artery (PA) coupling. While the traditional TAPSE/PASP ratio is widely used, the ratio of RV-FWLS/PASP serves as a highly sensitive, non-invasive surrogate for RV-PA coupling. A depressed RV-FWLS/PASP ratio indicates that the RV is failing to contract adequately against its afterload (mechanical uncoupling), allowing clinicians to identify latent right heart failure and advanced pulmonary vascular involvement during diagnostic workups [46].

### 4.2. Prognostic Significance of RV Strain in HFpEF

Impaired RV strain holds a powerful prognostic role in patients with HFpEF, correlating closely with elevated pulmonary artery pressures, worsening left ventricular diastolic dysfunction, and increased natriuretic peptide levels [50]. Chronically reduced RV-FWLS and depressed RV-FWLS/PASP coupling ratios are powerful independent predictors of adverse outcomes, strongly associated with higher rates of heart failure hospitalizations and all-cause mortality [51,52].

Mechanistically, this RV-PA uncoupling and impaired RV strain are principal drivers of exercise intolerance, the clinical hallmark of HFpEF. During exertion, the normal surge in venous return demands an increase in RV stroke volume. In HFpEF patients with impaired RV strain or latent RV-PA uncoupling, the right heart cannot accommodate this sudden hemodynamic load. This results in acute backward failure, profound hepatic and systemic venous congestion, and an inability to augment forward cardiac output. Clinically, this mechanical failure translates directly to severe exertional dyspnoea, reduced functional capacity, and heightened risk for acute decompensation events.

### 4.3. Right Ventricle Strain in HFpEF for Therapeutic-Monitoring

Evaluating RV-FWLS offers significant potential for monitoring therapeutic responses and right heart remodelling. Interventions that effectively lower left-sided filling pressures consequently alleviate pulmonary venous congestion and reduce RV afterload. Serial tracking of RV-FWLS and the RV-FWLS/PASP ratio allows clinicians to non-invasively monitor the stabilization or recovery of the right heart-pulmonary vasculature unit, offering a sensitive means to titrate therapies targeting congestion or body weight.

Despite its promising clinical utility, RV strain assessment remains limited by technical challenges related to RV geometry, image quality, and inter-vendor variability. Technically, the assessment of RV-FWLS is hindered by the RV thin myocardium, complex crescentic anatomy, and high sensitivity to near-field reverberation artefacts and acoustic dropouts. Furthermore, tracking quality depends heavily on obtaining an optimal RV-focused apical 4-chamber. Nevertheless, current evidence supports RV strain as a sensitive marker of right ventricular dysfunction and an important prognostic and monitoring tool in patients with HFpEF, even though it still needs further investigation and this technique is currently only applicable to medium-to-high-risk HFpEF patients complicated with pulmonary hypertension and is not recommended for routine screening in all patients at present.

## 5. Technical Limitations, Gaps in Knowledge and Future Perspectives

Although STE demonstrates considerable clinical value in HFpEF, some critical technical limitations and future directions must be acknowledged.

### 5.1. Technical Limitations

STE remains highly dependent on image quality. Poor endocardial border definition, acoustic dropouts, and suboptimal frame rates (ideally maintained between 40 and 80 frames per second) can cause tracking algorithms to fail or artificially underestimate strain values. Additionally, statistically significant vendor variability and intervendor reproducibility should be considered when implementing in a widespread clinical context and performing serial studies, as highlighted in the EACVI/ASE Inter-Vendor comparison study [53], even though LV-GLS measurements from companies that provide contemporary semi-automated clinical software and recently developed fully automated software for dedicated LA strain analysis have become more consistent in recent years, demonstrating excellent inter- and intra-operator reproducibility [54,55]. Furthermore, despite being more robust than LVEF, myocardial strain is fundamentally dependent on loading conditions. Acute shifts in afterload or significant changes in preload will modify the absolute strain magnitude [56], requiring clinicians to interpret strain values alongside the patient’s immediate hemodynamic state. Finally, the clinical utility of these metrics is hindered by a lack of universally accepted cut-offs, with normative thresholds defining definitive impairment versus borderline physiology remaining fluid across registries, societies, and software vendors, complicating the integration of multi-chamber strain into rigid diagnostic algorithms.

### 5.2. Clinical Diagnosis and Treatment

**Figure 5:**
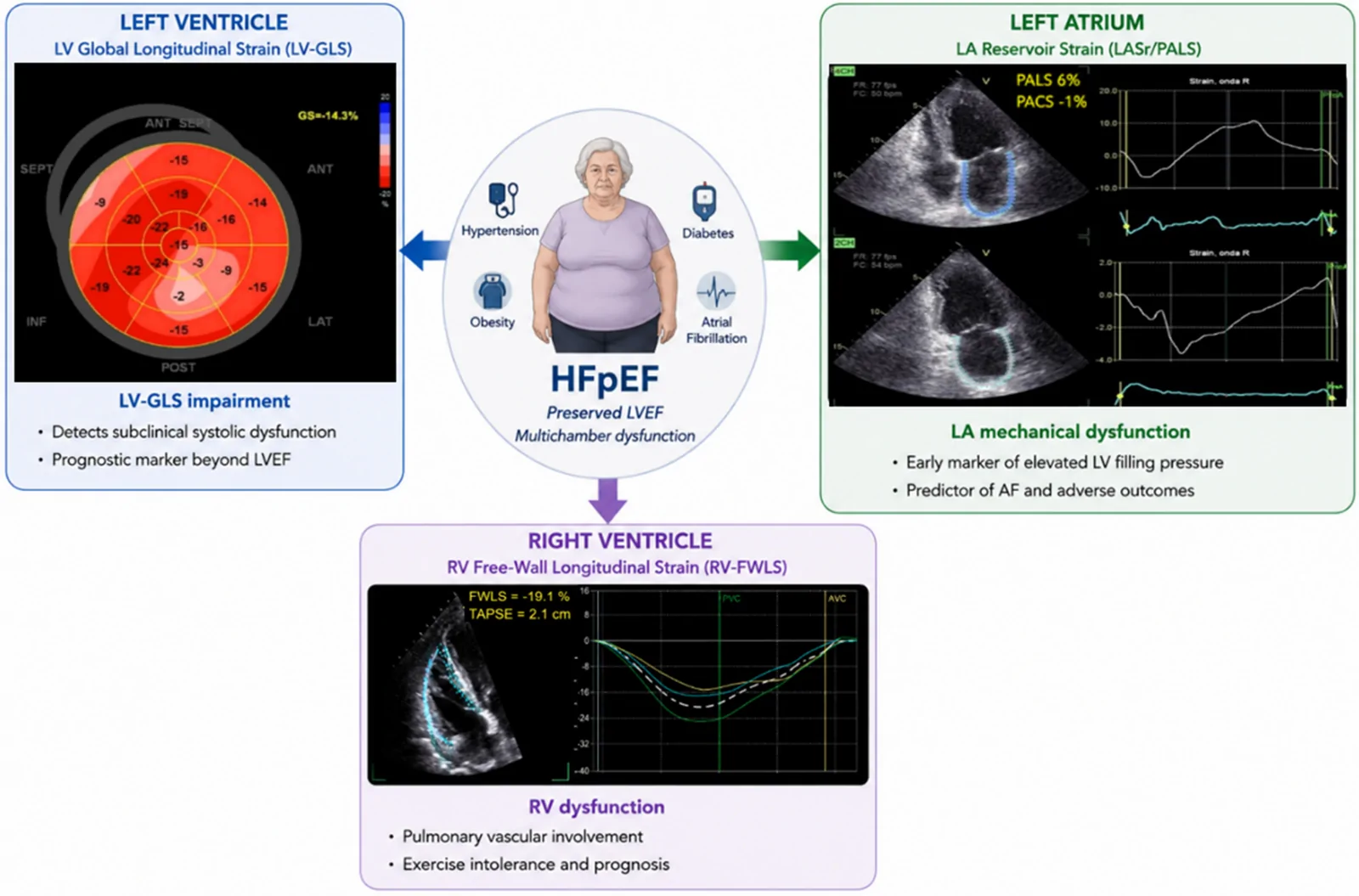
Use of speckle tracking in patients at risk of HFpEF for early diagnosis and prognostic assessment.

Current evidence supports the integration of STE into comprehensive echocardiographic evaluation to improve diagnostic accuracy, provide prognostic information, assist in risk stratification, and potentially guide timing of therapeutic interventions in HFpEF (Figure 5). Overall, strain echocardiography provides a comprehensive assessment of the multi-chamber involvement characterizing HFpEF and may contribute to a more personalized diagnostic and therapeutic approach according to multi-chamber STE cut-off values (Table 2), as suggested by the ASE/EACVI Consensus to identify high-risk patients when integrated with clinical, instrumental, and biomarker parameters [57].

LV-GLS should be incorporated into the routine evaluation of suspected HFpEF, as recommended by the HFA-PEFF diagnostic algorithm, to improve detection of subtle LV systolic dysfunction despite preserved LVEF. Beyond diagnosis, serial assessment of LV-GLS may represent a useful tool for monitoring disease progression and therapeutic response.

Among strain-derived parameters, LA strain appears particularly promising for clinical implementation. Given its ability to detect early LA dysfunction before structural remodelling occurs, PALS should be integrated in the evaluation of patients with unexplained dyspnoea to differentiate cardiac from non-cardiac causes and to identify elevated LV filling pressures when conventional echocardiographic parameters are inconclusive. Moreover, reflecting atrial cardiomyopathy severity, LA strain may refine risk stratification for incident atrial fibrillation and thromboembolic events.

Importantly, clinicians should consider the load-dependence of strain parameters, which are therefore limited in HFpEF in distinguishing intrinsic atrial myopathy from occult, intermittent, or exercise-induced elevation of left ventricular filling pressure. 

Finally, RV-FWLS has emerged as a sensitive marker of early RV involvement in HFpEF, reflecting the impact of pulmonary vascular disease and ventricular interdependence.

### 5.3. Interventional Trials

Despite its established prognostic value and potential utility, further prospective evidence and dedicated interventional trials are required before multi-chamber strain parameters, such as LV-GLS for treatment guidance and RV strain for therapeutic decision-making, can be systematically incorporated into routine clinical algorithms to directly guide treatment strategies.

### 5.4. Special Populations

Rhythm irregularities, most notably AF, can present a challenge to strain software. Cycle-length variability across irregular beats leads to inconsistent diastolic filling times and varying contractile forces. For LV and RV tracking, this necessitates averaging measurements across 5 to 10 consecutive cycles. For LA strain in AF, PACS is completely lost, and PALS values are intrinsically altered, necessitating unique, rhythm-specific reference frameworks. Nonetheless, in patients with established AF, assessment of LA mechanical function may provide incremental information beyond traditional clinical scores, such as CHA_2_DS_2_-VASc, potentially helping decision-making regarding anticoagulation in selected patients with borderline embolic risk.

## 6. Conclusions

The increasing prevalence of HFpEF, together with its complex and heterogeneous pathophysiology, continues to represent a major challenge for early diagnosis, risk stratification, and patient management. In the current era of disease-modifying therapies that extend beyond symptomatic relief and improve clinical outcomes, the identification of sensitive imaging markers able to detect early myocardial dysfunction and guide a more personalized approach has become increasingly relevant.

In this context, STE has emerged as a valuable tool for a comprehensive multi-chamber assessment of HFpEF. In recent years, many studies have suggested that combining standard echocardiographic evaluation with other advanced echocardiographic parameters, including LV strain, left atrial strain and right ventricular strain, may further enhance diagnostic and prognostic assessment in HFpEF. This multimodal approach reflects the systemic and multifactorial nature of the syndrome, in which ventricular, atrial, pulmonary, and vascular dysfunction coexist and contribute to adverse outcomes.

Current data strongly support the role of advanced echocardiographic methods as a sensitive marker of myocardial dysfunction and an important tool for diagnosis, risk stratification and prognosis in HFpEF patients, reinforcing the importance of its implementation in routine clinical practice.

## Figures and Tables

**Table 1:** LV-GLS patterns in different HFpEF phenocopies.

Phenocopy	Absolute LV GLS	Typical Strain Distribution Pattern	Key Strain Index/Diagnostic Clue	Pathophysiological Mechanism
Primary HFpEF	Mild to moderate reduction (typically −14% to −17%)	Diffuse, uniform impairment across all myocardial segments.	Relative Apical Sparing Ratio (RASR) <1.0; correlates broadly with metabolic comorbidities and hypertension.	Diffuse subendocardial remodeling, interstitial fibrosis, and coronary microvascular dysfunction.
Cardiac Amyloidosis	Severe reduction (frequently >−12%)	Classic ‘Apical Sparing’ pattern (severely depressed basal/mid-ventricular strain with preserved apex).	Relative Apical Sparing Ratio (RASR) >1.0; classic ‘cherry-on-top’ appearance on bullseye plot.	Infiltration of amyloid fibrils into the myocardial interstitium, causing restrictive expansion and apical sparing.
Hypertrophic Cardiomyopathy	Moderate to severe reduction	Segmental or regional impairment strictly corresponding to sites of maximal hypertrophy.	Heterogeneous ‘patchy’ look on bullseye plot; adjacent non-hypertrophied segments display normal strain.	Myofibrillar disarray, focal hypertrophy, and replacement fibrosis disrupting regional contraction mechanics.
Fabry Disease	Mild to moderate reduction	Localized loss of longitudinal strain specifically in the basal posterolateral (or posteromedial) wall.	Early regional loss matching areas of mid-wall late gadolinium enhancement (LGE) on cardiac MRI.	Lysosomal accumulation of glycosphingolipids leading to progressive hypertrophy, inflammation, and localized fibrosis.

**Table 2:** Multi-chamber strain cut-off values from ASE/EACVI consensus.

Chamber and Parameter	Normal Reference Range	Impairment/High Risk
LV Global Longitudinal Strain (LV GLS)	<−18%	>−16%
LA Reservoir Strain (LARS)	>35%	<23%
RV Strain (RV-FWS)	−29.0% ± 4.5%	>−20% in men and >−21% in women

## Data Availability

No new data were created or analyzed in this study. Data sharing is not applicable to this article.

## References

[1] McDonagh T.A., Metra M., Adamo M., Gardner R.S., Baumbach A., Böhm M., Burri H., Butler J., Čelutkienė J., Chioncel O. (2021). 2021 ESC Guidelines for the diagnosis and treatment of acute and chronic heart failure: Developed by the Task Force for the diagnosis and treatment of acute and chronic heart failure of the European Society of Cardiology (ESC) with the special contribution of the Heart Failure Association (HFA) of the ESC. Eur. Heart J..

[2] Borlaug B.A., Sharma K., Shah S.J., Ho J.E. (2023). Heart Failure with Preserved Ejection Fraction: Scientific Statement. JACC.

[3] Sanna G.D., Canonico M.E., Santoro C., Esposito R., Masia S.L., Galderisi M., Parodi G., Nihoyannopoulos P. (2021). Echocardiographic Longitudinal Strain Analysis in Heart Failure: Real Usefulness for Clinical Management Beyond Diagnostic Value and Prognostic Correlations? A Comprehensive Review. Curr. Heart Fail Rep..

[4] Marwick T.H. (2018). Ejection Fraction Pros and Cons: JACC State-of-the-Art Review. J. Am. Coll. Cardiol..

[5] Hiebert J.B., Vacek J., Shah Z., Rahman F., Pierce J.D. (2019). Use of speckle tracking to assess heart failure with preserved ejection fraction. J. Cardiol..

[6] Pastorini G., Anastasio F., Feola M. (2023). What Strain Analysis Adds to Diagnosis and Prognosis in Heart Failure Patients. J. Clin. Med..

[7] Hamada-Harimura Y., Seo Y., Ishizu T., Nishi I., Machino-Ohtsuka T., Yamamoto M., Sugano A., Sato K., Sai S., Obara K. (2018). Incremental Prognostic Value of Right Ventricular Strain in Patients with Acute Decompensated Heart Failure. Circ. Cardiovasc. Imaging.

[8] Medvedofsky D., Kebed K., Laffin L., Stone J., Addetia K., Lang R.M., Mor-Avi V. (2017). Reproducibility and experience dependence of echocardiographic indices of left ventricular function: Side-by-side comparison of global longitudinal strain and ejection fraction. Echocardiography.

[9] Wang J., Khoury D.S., Yue Y., Torre-Amione G., Nagueh S.F. (2008). Preserved left ventricular twist and circumferential deformation, but depressed longitudinal and radial deformation in patients with diastolic heart failure. Eur. Heart J..

[10] Lee J.H., Park J.H. (2025). Clinical Applications of Speckle-Tracking Echocardiography in Heart Failure: From Diagnosis to Prognostication. Int. J. Heart Fail.

[11] Morris D.A., Ma X.X., Belyavskiy E., Aravind Kumar R., Kropf M., Kraft R., Frydas A., Osmanoglou E., Marquez E., Donal E. (2017). Left ventricular longitudinal systolic function analysed by 2D speckle-tracking echocardiography in heart failure with preserved ejection fraction: A meta-analysis. Open Heart.

[12] Pieske B., Tschöpe C., A de Boer R., Fraser A.G., Anker S.D., Donal E., Edelmann F., Fu M., Guazzi M., Lam C.S.P. (2019). How to diagnose heart failure with preserved ejection fraction: The HFA-PEFF diagnostic algorithm. Eur. Heart J..

[13] Taylor C.J., Taylor K.S., Jones N.R., Ordóñez-Mena J.M., Bayes-Genis A., Hobbs F.D.R. (2025). Age-adjusted natriuretic peptide thresholds for a diagnosis of heart failure in the community: Diagnostic accuracy study. ESC Heart Fail..

[14] de Gregorio C., Trimarchi G., Faro D.C., De Gaetano F., Campisi M., Losi V., Zito C., Tamburino C., Di Bella G., Monte I.P. (2023). Myocardial Work Appraisal in Transthyretin Cardiac Amyloidosis and Nonobstructive Hypertrophic Cardiomyopathy. Am. J. Cardiol..

[15] de Gregorio C., Trimarchi G., Faro D.C., Poleggi C., Teresi L., De Gaetano F., Zito C., Lofrumento F., Koniari I., Licordari R. (2024). Systemic Vascular Resistance and Myocardial Work Analysis in Hypertrophic Cardiomyopathy and Transthyretin Cardiac Amyloidosis with Preserved Left Ventricular Ejection Fraction. J. Clin. Med..

[16] Lin Y., Xie M., Zhang L., Zhang Y., Zhang P., Chen X., Ji M., Gao L., He Q., Wu Z. (2025). Prognostic Value of LV Global Longitudinal Strain by 2D and 3D Speckle-Tracking Echocardiography in Patients with HFpEF. Circ. Cardiovasc. Imaging.

[17] Biering-Sørensen T., Biering-Sørensen S.R., Olsen F.J., Sengeløv M., Jørgensen P.G., Mogelvang R., Shah A.M., Jensen J.S. (2017). Global Longitudinal Strain by Echocardiography Predicts Long-Term Risk of Cardiovascular Morbidity and Mortality in a Low-Risk General Population: The Copenhagen City Heart Study. Circ. Cardiovasc. Imaging.

[18] Xu X., Liu H., Chen R. (2026). Long-term SGLT2 inhibitor therapy improves myocardial strain and diastolic function in HFpEF: A retrospective study linking functional recovery to reduced myocardial fibrosis. Front. Cardiovasc. Med..

[19] Thomas L., Marwick T.H., Popescu B.A., Donal E., Badano L.P. (2019). Left Atrial Structure and Function, and Left Ventricular Diastolic Dysfunction: JACC State-of-the-Art Review. J. Am. Coll. Cardiol..

[20] Robinson S., Ring L., Oxborough D., Harkness A., Bennett S., Rana B., Sutaria N., Lo Giudice F., Shun-Shin M., Paton M. (2024). The assessment of left ventricular diastolic function: Guidance and recommendations from the British Society of Echocardiography. Echo Res. Pract..

[21] Gan G.C.H., Ferkh A., Boyd A., Thomas L. (2018). Left atrial function: Evaluation by strain analysis. Cardiovasc. Diagn. Ther..

[22] Kagami K., Harada T., Yuasa N., Saito Y., Sorimachi H., Murakami F., Naito A., Tani Y., Kato T., Wada N. (2024). Impaired Left Atrial Reserve Function in Heart Failure with Preserved Ejection Fraction. Circ. Cardiovasc. Imaging.

[23] Maffeis C., Morris D.A., Belyavskiy E., Kropf M., Radhakrishnan A.K., Zach V., Rozados da Conceicao C., Trippel T.D., Pieske-Kraigher E., Rossi A. (2021). Left atrial function and maximal exercise capacity in heart failure with preserved and mid-range ejection fraction. ESC Heart Fail..

[24] Inoue K., Khan F.H., Remme E.W., Ohte N., García-Izquierdo E., Chetrit M., Moñivas-Palomero V., Mingo-Santos S., Andersen Ø.S., Gude E. (2021). Determinants of left atrial reservoir and pump strain and use of atrial strain for evaluation of left ventricular filling pressure. Eur. Heart J. Cardiovasc. Imaging.

[25] Lababidi H., Rahi W., Smiseth O.A., Billick K., Inoue K., Khan F.H., Andersen Ø.S., García-Izquierdo E., Ha J.W., Ohte N. (2025). New Algorithm for Estimating Left Ventricular Filling Pressure by Echocardiography. Circulation.

[26] Smiseth O.A., Morris D.A., Cardim N., Cikes M., Delgado V., Donal E., A Flachskampf F., Galderisi M., Gerber B.L., Gimelli A. (2022). Multimodality imaging in patients with heart failure and preserved ejection fraction: An expert consensus document of the European Association of Cardiovascular Imaging. Eur. Heart J. Cardiovasc. Imaging.

[27] Miyata M. (2025). Arterial stiffness and left atrial function. Hypertens. Res..

[28] Mondillo S., Cameli M., Caputo M.L., Lisi M., Palmerini E., Padeletti M., Ballo P. (2011). Early detection of left atrial strain abnormalities by speckle-tracking in hypertensive and diabetic patients with normal left atrial size. J. Am. Soc. Echocardiogr..

[29] Muranaka A., Yuda S., Tsuchihashi K., Hashimoto A., Nakata T., Miura T., Tsuzuki M., Wakabayashi C., Watanabe N., Shimamoto K. (2009). Quantitative assessment of left ventricular and left atrial functions by strain rate imaging in diabetic patients with and without hypertension. Echocardiography.

[30] Miyoshi H., Oishi Y., Mizuguchi Y., Iuchi A., Nagase N., Ara N., Oki T. (2014). Contribution of obesity to left atrial and left ventricular dysfunction in asymptomatic patients with hypertension: A two-dimensional speckle-tracking echocardiographic study. J. Am. Soc. Hypertens..

[31] Chirinos J.A., Sardana M., Satija V., Gillebert T.C., De Buyzere M.L., Chahwala J., De Bacquer D., Segers P., Rietzschel E.R., Asklepios investigators (2019). Effect of Obesity on Left Atrial Strain in Persons Aged 35-55 Years (The Asklepios Study). Am. J. Cardiol..

[32] Venkateshvaran A., Oktay Tureli H., Ljung Faxén U., Lund L.H., Tossavainen E., Lindqvist P. (2022). Left atrial reservoir strain improves diagnostic accuracy of the 2016 ASE/EACVI diastolic algorithm in patients with preserved left ventricular ejection fraction: Insights from the KARUM haemodynamic database. Eur. Heart J.-Cardiovasc. Imaging.

[33] Backhaus S.J., Schmermund B.N., Rieth A.J., Rademann M., Kriechbaum S.D., Wolter J.S., Wiedenroth C.B., Schulz A., Lange T., Treiber J.M. (2025). Calculation of pulmonary capillary wedge pressure including left atrial function is superior to morphology alone and accurately identifies elevated filling pressures in left heart disease. J. Cardiovasc. Magn. Reson..

[34] Kagami K., Harada T., Yuasa N., Tani Y., Murakami F., Saito Y., Naito A., Okuno T., Kato T., Takama N. (2025). A scoring system for diagnosing heart failure with preserved ejection fraction based on exercise echocardiography. Eur. Heart J. Cardiovasc. Imaging.

[35] Rausch K., Scalia G.M., Sato K., Edwards N., Lam A.K., Platts D.G., Chan J. (2021). Left atrial strain imaging differentiates cardiac amyloidosis and hypertensive heart disease. Int. J. Cardiovasc. Imaging.

[36] Monte I.P., Faro D.C., Trimarchi G., de Gaetano F., Campisi M., Losi V., Teresi L., Di Bella G., Tamburino C., de Gregorio C. (2023). Left Atrial Strain Imaging by Speckle Tracking Echocardiography: The Supportive Diagnostic Value in Cardiac Amyloidosis and Hypertrophic Cardiomyopathy. J. Cardiovasc. Dev. Dis..

[37] Khan M.S., Memon M.M., Murad M.H., Vaduganathan M., Greene S.J., Hall M., Triposkiadis F., Lam C.S.P., Shah A.M., Butler J. (2020). Left atrial function in heart failure with preserved ejection fraction: A systematic review and meta-analysis. Eur. J. Heart Fail.

[38] Mandoli G.E., Cameli M., Pastore M.C., Benfari G., Malagoli A., D’Andrea A., Sperlongano S., Bandera F., Esposito R., Santoro C. (2023). Speckle tracking echocardiography in early disease stages: A therapy modifier?. J. Cardiovasc. Med..

[39] Santos A.B., Kraigher-Krainer E., Gupta D.K., Claggett B., Zile M.R., Pieske B., Voors A.A., Lefkowitz M., Bransford T., Shi V. (2014). Impaired left atrial function in heart failure with preserved ejection fraction. Eur. J. Heart Fail.

[40] Telles F., Nanayakkara S., Evans S., Patel H.C., Mariani J.A., Vizi D., William J., Marwick T.H., Kaye D.M. (2019). Impaired left atrial strain predicts abnormal exercise haemodynamics in heart failure with preserved ejection fraction. Eur. J. Heart Fail..

[41] Sonaglioni A., Nicolosi G.L. (2025). Left Atrial Reservoir Strain in Cardiovascular and Systemic Disease: Advances and Clinical Applications from Physiology to Practice. Rev. Cardiovasc. Med..

[42] Olsen F.J., Diederichsen S.Z., Jørgensen P.G., Jensen M.T., Dahl A., Landler N.E., Graff C., Brandes A., Krieger D., Haugan K. (2024). Left Atrial Strain Predicts Subclinical Atrial Fibrillation Detected by Long-term Continuous Monitoring in Elderly High-Risk Individuals. Circ. Cardiovasc. Imaging.

[43] Obokata M., Negishi K., Kurosawa K., Tateno R., Tange S., Arai M., Amano M., Kurabayashi M. (2014). Left atrial strain provides incremental value for embolism risk stratification over CHA_2_DS_2_-VASc score and indicates prognostic impact in patients with atrial fibrillation. J. Am. Soc. Echocardiogr..

[44] Di Salvo G., Caso P., Lo Piccolo R., Fusco A., Martiniello A.R., Russo M.G., D’Onofrio A., Severino S., Calabró P., Pacileo G. (2005). Atrial myocardial deformation properties predict maintenance of sinus rhythm after external cardioversion of recent-onset lone atrial fibrillation: A color Doppler myocardial imaging and transthoracic and transesophageal echocardiographic study. Circulation.

[45] Barilli M., Mandoli G.E., Sisti N., Dokollari A., Ghionzoli N., Soliman-Aboumarie H., D’Ascenzi F., Focardi M., Cavigli L., Pastore M.C. (2024). Potential Role of Left Atrial Strain to Predict Atrial Fibrillation Recurrence after Catheter Ablation Therapy: A Clinical and Systematic Review. J. Cardiovasc. Dev. Dis..

[46] Lejeune S., Roy C., Ciocea V., Slimani A., de Meester C., Amzulescu M., Pasquet A., Vancraeynest D., Beauloye C., Vanoverschelde J.L. (2020). Right Ventricular Global Longitudinal Strain and Outcomes in Heart Failure with Preserved Ejection Fraction. J. Am. Soc. Echocardiogr..

[47] Hubesch G., Dewachter C., Chomette L., Hupkens E., Jespers P., Vegh G., Doppler M., Sheikh Mohammad U., Thiriard A., Remmelink M. (2024). Early Alteration of Right Ventricle-Pulmonary Artery Coupling in Experimental Heart Failure with Preserved Ejection Fraction. J. Am. Heart Assoc..

[48] Ji M., Wu W., He L., Gao L., Zhang Y., Lin Y., Qian M., Wang J., Zhang L., Xie M. (2022). Right Ventricular Longitudinal Strain in Patients with Heart Failure. Diagnostics.

[49] Tadic M., Nita N., Schneider L., Kersten J., Buckert D., Gonska B., Scharnbeck D., Reichart C., Belyavskiy E., Cuspidi C. (2021). The Predictive Value of Right Ventricular Longitudinal Strain in Pulmonary Hypertension, Heart Failure, and Valvular Diseases. Front Cardiovasc. Med..

[50] Carlsson M., Ugander M., Heiberg E., Arheden H. (2007). The quantitative relationship between longitudinal and radial function in left, right, and total heart pumping in humans. Am. J. Physiol. Heart Circ. Physiol..

[51] Amsallem M., Mercier O., Kobayashi Y., Moneghetti K., Haddad F. (2018). Forgotten No More: A Focused Update on the Right Ventricle in Cardiovascular Disease. JACC Heart Fail..

[52] Backhaus S.J., Schulz A., Lange T., Rösel S.F., Schmidt-Schweda L.S., Kutty S., Kowallick J.T., Treiber J., Rolf A., Sossalla S. (2025). Insights from serial cardiovascular magnetic resonance imaging show early progress in diastolic dysfunction relates to impaired right ventricular deformation. Sci. Rep..

[53] Farsalinos K.E., Daraban A.M., Ünlü S., Thomas J.D., Badano L.P., Voigt J.-U. (2015). Head-to-Head Comparison of Global Longitudinal Strain Measurements among Nine Different Vendors: The EACVI/ASE Inter-Vendor Comparison Study. J. Am. Soc. Echocardiogr..

[54] Balinisteanu A., Duchenne J., Puvrez A., Wouters L., Bézy S., Youssef A., Minten L., Bekhuis Y., Van Langenhoven L., Papangelopoulou K. (2025). Vendor differences in 2D-speckle tracking global longitudinal strain: An update on a 10-year standardization effort. Eur. Heart J. Cardiovasc. Imaging.

[55] Mandoli G.E., Pastore M.C., Procopio M.C., Pica A., Vigna M., Benfari G., Diviggiano E.E., Martini L., Lunghetti S., Focardi M. (2024). Unveiling the reliability of left atrial strain measurement: A dedicated speckle tracking software perspective in controls and cases. Eur. Heart J. Imaging Methods Pract..

[56] Mihos C.G., Liu J.E., Anderson K.M., Pernetz M.A., O’Driscoll J.M., Aurigemma G.P., Ujueta F., Wessly P., on behalf of the American Heart Association Council on Peripheral Vascular Disease; Council on Cardiovascular and Stroke Nursing; and Council on Clinical Cardiology (2025). Speckle-Tracking Strain Echocardiography for the Assessment of Left Ventricular Structure and Function: A Scientific Statement from the American Heart Association. Circulation.

[57] Thomas J.D., Edvardsen T., Abraham T., Appadurai V., Badano L., Banchs J., Cho G.Y., Cosyns B., Delgado V., Donal E. (2026). Clinical Applications of Strain Echocardiography: A Clinical Consensus Statement from the American Society of Echocardiography Developed in Collaboration with the European Association of Cardiovascular Imaging of the European Society of Cardiology. Eur. Heart J. Cardiovasc. Imaging.

